# Exploring Tiny Homes as an Affordable Housing Strategy to Ameliorate Homelessness: A Case Study of the Dwellings in Tallahassee, FL

**DOI:** 10.3390/ijerph17020661

**Published:** 2020-01-20

**Authors:** April Jackson, Bridget Callea, Nicholas Stampar, Abigail Sanders, Alberto De Los Rios, Jake Pierce

**Affiliations:** Department of Urban and Regional Planning, Florida State University, Tallahassee, FL 23206, USA; bncallea@aol.com (B.C.); nstampar@gmail.com (N.S.); ags14@my.fsu.edu (A.S.); jad13@my.fsu.edu (A.D.L.R.); jakepierce818@gmail.com (J.P.)

**Keywords:** tiny homes, homelessness, affordable housing, planning, new urbanism

## Abstract

An emerging strategy to combat homelessness is the development of “tiny homes”. However, the advent of tiny homes as a new form of housing intervention raises a number of questions about their intentions, efficacy, and policy feasibility. This paper seeks to understand the strategies used by stakeholders to plan, design, and implement a tiny home community to meet the needs of those experiencing homelessness, and to understand where these plans were effective and where challenges arose in meeting the intended project goals. Utilizing the recent development of Tallahassee’s “The Dwellings” project as a case study, we examine how the community was planned, resident experiences, and constraints to implementing a tiny home development. We use qualitative methods that include interviews with stakeholders who were integral to the planning and development process. Our findings highlight how funding constraints and NIMBYism (Not in My Backyard-ism) stymied stakeholder efforts to achieve equity and affordability at The Dwellings, resulting in the inability to achieve project aims of developing housing that serves the homeless population. We conclude by offering some observations and lessons learned for future research on tiny homes as a solution to ameliorate homelessness.

## 1. Introduction

Attempts to solve homelessness have ranged in strategy and theory, with a multitude of approaches that have varying degrees of effectiveness. An emerging strategy to combat homelessness is the development of transitional housing and even, more recently, the construction of “tiny homes”. Morrison and Hammer [1] define tiny homes as any structure that is less than 400 square feet in area, excluding lofts. Tiny homes became mainstream as affordable housing alternatives when they were used as temporary shelters following Hurricane Katrina [2,3]. This affordable housing alternative was later used as a strategy to combat issues of homelessness throughout the United States [4,5,6]. With the decrease in the supply of affordable housing and more residents being cost burdened, or spending 30% or more of their income on housing, tiny homes are a popular trend not only to provide cheaper housing options, but also to offer traditional neighborhood designs that are sustainable and support minimalist living trends [7]. However, the advent of tiny homes as a new form of strategic housing intervention raises a number of questions about the intentions, efficacy, and policy feasibility of these minimalist structures. 

The general efficacy of tiny homes as a viable housing intervention to meet the needs of those experiencing homelessness remains an understudied issue [8,9,10]. This paper seeks to understand the strategies used by stakeholders to plan, design, and implement a tiny home community to meet the needs of those experiencing homelessness, and to understand where these plans were effective and where there were challenges in meeting the intended project goals. Utilizing the recent development of Tallahassee’s “The Dwellings” project as a case study, we examine how the community was planned, resident experiences, and constraints to implementing a tiny home development aimed at ameliorating homelessness in Tallahassee. Our main research question is “How do The Dwellings meet the needs of filling the affordable housing gap for those experiencing homelessness?” We use qualitative methods that include interviews with twelve stakeholders that were integral to the planning and development process, including staff and residents of The Dwellings, consultants involved in the design process, as well as outside agencies and municipal planners. The data collection and analyses were conducted between January 2018–June 2018. Our findings show that there are a number of hurdles involved with developing tiny homes for the homeless, including community pushback, funding sources, and development regulations. However, with the appropriate funding in place, strategic decision-making, collaboration, and support services, tiny homes may be a useful tactic for reducing homelessness. Overall, we illustrate the challenges faced to provide affordable housing within the context of a lack of comprehensive local, state, and federal policies that limit the non-profit and private sectors’ ability to maintain housing affordability, particularly for low barrier housing.

This article begins with a review of the literature on homelessness and tiny homes as an affordable housing strategy for those experiencing homelessness. Next, we discuss The Dwellings tiny home community and then offer findings that highlight the participation and collaboration activities undertaken, resident experiences, and project constraints faced by stakeholders during implementation. Our findings highlight how funding constraints and NIMBYism (Not in My Backyard-ism) stymied stakeholder efforts to achieve equity and affordability at The Dwellings, which resulted in the inability to achieve project aims of developing housing that serves the homeless population. We conclude by offering some observations and lessons learned for future research on tiny homes as a solution to ameliorate homelessness.

## 2. Context for Issues of Homelessness

It is essential to understand the need for affordable housing by providing the context of the causes that lead to housing insecurity, current needs for those experiencing homelessness, and strategies aimed at providing transitional housing, such as tiny homes, as an affordable housing solution. 

Approximately 150,000 families with 330,000 children stay in homeless shelters each year in the United States [11]. However, there are millions more who are housing insecure and at risk of homelessness. According to a national estimate taken in 2017, there were approximately 17 people experiencing homelessness per every 10,000 people in the general population [7,12]. In 2017, the majority of the homeless population lived in some form of shelter or transitional housing; and yet, approximately 34% lived in places unfit for human habitation, such as on the street or in abandoned buildings. The majority of the homeless are single individuals (66.7%), while the remaining are families. Among the homeless populations, ethnic minorities, primarily African American (40%) and Hispanic/Latino (21%), are at higher risk of homelessness and comprise a larger portion of the long-term homeless population. These individuals face higher susceptibility based on institutionalized racism and the likelihood that they do not have access to supportive services that can prevent homelessness [13,14,15]. Additionally, 7.2% were veterans and 7.4% were unaccompanied children and young adults [16]. 

Between 2007 and 2017, overall homelessness decreased 14.4%, with the most drastic decreases occurring for veterans (34.3%), individuals experiencing chronic homelessness (27.4%), and people living in unsheltered locations (24.6%) [12]. However, many people with low incomes are at risk of homelessness, broadly because of a lack of affordable housing [17]. The number of poor renter households experiencing a severe housing cost burden in 2016 (paying more than 50% of their income toward housing) was 6,902,060. This is 20.8% greater than in 2007. An estimated additional 4,609,826 people in poor households were living “doubled up” with family and friends in 2016. This is also much higher (30% greater) than in 2007 and represents one of the most common prior-living situations for people who become homeless [16]. The vast amount of people affected by this crisis emphasizes the need for adequate affordable housing. 

The literature points to a variety of reasons why people face homelessness or housing insecurity [18,19]. Wright and Rubin [20] suggest homelessness is, by definition, a lack of housing, but the United States seems to have an adequate supply of market rate housing but lacks enough affordable housing [20,21]. The National Low Income Housing Coalition (NLIHC) reported in 2017 that the United States currently has a shortage of 7.4 million affordable and available rental units. Among the individuals and families most in need, 11.4 million households are extremely low-income (ELI), falling at or below the poverty guideline, or 30% of the area’s adjusted median income (AMI), whichever is higher [22]. ELI households account for almost one-third of U.S. renter households and nearly 10% of all existing households. The inability to afford housing perpetuates the common perception of financial instability as the reason for homelessness [23]. However, Lindblom [24] argues extreme poverty is not the primary predictor of homelessness. Several scholars argue that secondary characteristics, such as youth, alcohol and other drug problems, institutional histories, and weak support networks provide better predictors for those most at risk for homelessness [24,25,26]. These secondary characteristics require a more comprehensive approach than merely providing housing [27]. Often more traditional solutions to homelessness, such as emergency shelters and transitional housing, do not offer comprehensive needs assessment plans. 

Shelters often have restrictions on the length of time people may stay, which contributes to long-term issues [28]. Further, some shelters do not provide resources to assist people in finding better opportunities, locking them into a cycle of need [29,30]. More specifically, those experiencing homelessness tend to exhibit high levels of mental illness, substance abuse, social estrangement, and deep poverty [20]. Homeless populations have reported unmet health needs, inability to obtain medical care, lack of access to prescription medications, inability to obtain eyeglasses, and lack of dental care. Predictors of these unmet needs include food insufficiency, vision impairment, out-of-home placement, and lack of health insurance [31]. 

Subsets of the homeless population may have even more specific needs based on their experiences [32]. For instance, homeless mothers have proven to have few economic resources, experience high rates of abuse and assault, and have psychosocial and physical health needs [33]. Tually and colleagues found that most mothers became homeless following domestic or neighbor abuse, or the breakdown of family relationships [34]. Guo, Slesnick, and Feng [35] explain that between 28% to 50% of homeless mothers report utilizing illicit drugs within one year of becoming homeless [36,37]. These estimates can be underrepresented by mothers who fear separation from their children and avoidance of mental health treatments [35,38]. Their perceptions of the services available were mixed. While some valued the support offered by staff and residents, the majority felt that there was a lack of resources to address their needs [39]. Researchers concluded that services need to work to address the multiple health, social, psychological, and housing needs of men [40]. 

Homeless youth are also at risk for physical and mental health problems stemming from a high likelihood of experiencing familial dysfunction, physical and sexual abuse, neglect, and substance abuse [41]. In recent years, large metropolitan areas have experienced a rampant increase in homeless youth. For instance, Seattle’s King County experienced a 700% increase of homeless youth between 2016 and 2017 [42]; San Diego’s homeless young adults increased by 39% in 2016 [43]. Among this increasing young adult population, up to 40 percent are part of the lesbian, gay, bisexual, transgender (LGBT+) community. Wiltz [43] notes that many of these young adults are sexually exploited in exchange for unstable housing, which negatively impacts physical, emotional, and social development and can result in students dropping out of school. Additionally, the LGBTQ+ population comprises 20–40% of homeless populations, while LGBTQ+ people are 5–10% of the North American population [44,45]. Moreover, there is limited research on the intersectionalities between LGBTQ+ identity and relationships between poverty, race and ethnicity, or gender [46].

Another susceptible homeless subpopulation is older adults, who are likely to have poor health, lack of social support, experience of longer durations of homelessness, lack of employment, and limited access to mental health treatment [15,47]. Fusaro and colleagues (2018) found that homeless non-Hispanic Blacks had the highest likelihood (one out of six) of lifetime homeless prevalence compared to Hispanics (one out of 12) and Whites (one out of 20).

### Tiny Homes as A Strategy To Ameliorate Homelessness

The increase in homelessness is attributed to social and economic policy. Dolbeare, Saraf, and Crowley [48] indicate that, in the past three decades, the federal housing assistance budget has dropped by nearly 50%. In conjunction with increased financial inequality, low-wage jobs, and rise in health care costs, over 50 million Americans cannot afford health care and many are unable to rent a two-bedroom apartment [22,49]. The National Health Care for the Homeless Council [50] notes that, despite multiple assistance programs, many do not address the structural causes of homelessness and thus have not suppressed its rise.

Preventative approaches such as accessible health care, better living wages, stronger social services, and enhanced housing affordability policies are oriented towards a more “universal approach” which targets the root causes of homelessness [28,51]. HUD’s 2005 report on preventing homelessness recommends to only employ processes shown to be effective in previous studies, which include provision of housing subsidies and supportive mental/social services; mediation in housing courts; cash assistance for mortgage or rent; and assisting in rapid exit from shelters. According to HUD, the main barriers to implementation are funding limits and lack of comprehensive research. Nicholas and Henwood [28] indicate that providing financial security and supportive employment are strategies that can ease transition into stable mainstream housing. Yet, these strategies lack efficacy and efficiency due to limited funding and the need for holistic community efforts. HUD claims that its practice Continuum of Care (CoC) engages communities in homelessness prevention efforts by requiring funds for prevention-oriented initiatives, which help to better identify vulnerable populations and focus more on primary prevention strategies [52]. 

Tiny homes pose a solution for housing shortages and affordability. However, tiny home developments face obstacles, including housing market and land use policies oriented to favor the demands of the wealthy, such as establishing a minimum household size and limiting the number of dwelling units per parcel [53,54]. Zoning, land use codes, and building regulations are barriers that hinder higher density development in urban spaces [3,6]. Specifically, tiny homes are often considered accessory dwelling units (ADUs) or secondary units considered small dwellings that may reside behind a single-family residential property [55]. Brown [9] suggests that local and state governments can facilitate living in smaller units by reshaping local codes and zoning requirements. Additionally, Wegmann and Chapple [55] support backyard cottages as a strategy to promote smart growth strategies. With greater flexibility in zoning, land use codes, and building regulations, tiny homeowners would avoid facing a multitude of challenges such as legality of occupancy, insurance acquisition, financing, and repair of their houses.

Evans [6] poses a framework for potential solutions to overcome barriers in tiny home community developments. The initial step consists of reconfiguring the social perception of small homes as undesirable, low-quality housing. Improved design guidelines and enhanced space functionality could make infill housing more appealing. This, however, must be accompanied with flexibility of affordability, especially when these homes cater to low-income residents and homeless transitional programs. More importantly, Evans [6] emphasizes that shifting away from traditional zoning practices, increasing density, and lowering minimum lot requirements could create a legal pathway for tiny homes to enter American society’s housing standards. Lastly, Talen [56] suggests form-based codes (FBCs) oriented to accommodate work/live environments and urban sprawl mitigation can provide sustainable design and/or practices to allow for more effective integration of mixed housing developments and increased density. 

Tiny homes provide a low-cost solution to create a greater supply of affordable housing. Culhane and Metraux [4] conclude “it is both more efficient and more humane to reallocate resources currently devoted to shelters.” Fisher et al. [30] and Rodriguez and Eidelman [57] agree that the transitional nature of many housing solutions does not adequately serve the populations seeking these services. Disinvestment in shelter services could also steer people toward using mainstream social welfare programs, which are more cost-effective and which focus more on chronic homelessness by providing permanent supportive housing [4]. The Homeless Research Institute [58] further supports Culhane’s ideas, as its primary goal is to address homelessness by promoting housing stability initiatives alongside comprehensive services to address mental health, income, etc. Further research supports this, finding the most critical factors of success cited by participants was their desire for stability. While definitions of stability varied by individual, common themes included living in their own residence, affordability, no family separation, and extended time [30].

Existing literature shows the need for new ways to address housing insecurity, as well as the importance of prevention-oriented policies [4,58]. In tiny home communities throughout the country, elements of implementation vary (such as size and price) (see Table 1).

Based on the existing literature, most tiny home communities are funded by non-profits and provide supportive services. However, the size, price, and target populations vary significantly. While a number of tiny home communities target veteran populations or those with significant barriers to housing, there are also examples of more generalized programs. Considering these divergent approaches and their broad geographic scope, the planning process and design range broadly. Many projects rely on continued support in the form of charitable donations and volunteers. Interestingly, there are a limited number of these projects in Florida. Notably, while there are proposed tiny home developments in cities such as Jacksonville and Orlando, no tiny home development in Florida is featured as successful. This may be due to state-level barriers, as Florida is known for its land development procedures that may maximize building footprints or limit densities. However, this was not examined in the scope of this research paper.

Existing literature on the link between traditional neighborhood design (TND) and homelessness is limited and largely falls into two categories: questions of New Urbanism’s general efficacy in pursuing goals of inclusivity, affordability, and diversity, and potential relationships between homelessness and health, and the various presumed effects of traditional neighborhood design. Links are drawn between purported outcomes of New Urbanism or TND and homelessness, health, and homeless integration. For instance, one study examined the impact of housing type, neighborhood characteristics, and lifestyles on the community integration of formerly homeless individuals with mental illnesses [59,60]. In the next section, we will describe the case of The Dwellings in Tallahassee, FL to provide the context of our research findings.

## 3. Background, Case Study Description, and Methods

There are currently 35 low income housing complexes in and around Tallahassee. Many are exclusively for those over the age of 55 or those with disabilities [61]. The Tallahassee Housing Authority (THA) [62] owns and operates over 541 apartments and scattered site homes throughout Leon County, where participants are responsible for paying approximately 30% of their household “adjusted” income. This program maintains a waiting list for residents hoping to receive access to housing. Additionally, the THA offers Housing Choice Vouchers that serve over 2000 families per month. However, the THA is currently not accepting applications to the voucher program, as the waiting list is closed. In terms of services specifically for the homeless, currently there are eight organizations in Tallahassee: City Walk Urban Mission, The Chelsea House, CARE Tallahassee, Wisdom’s Wellspring, The Kearney Center, Big Bend Homeless Coalition, Catholic Charities of Northwest Florida Emergency Services, and Making Miracles Group Home. Each of these offer different services for specific subpopulations, including transitional housing for pregnant women and those who have been incarcerated, and emergency housing for women with children. Further, each program offers different lengths of stay, limiting the amount of aid one can receive from each specific location [63]. The Kearney Center, or “the Shelter” is one of the largest operations and is a part of the CESC (Connecting Everyone to Second Chances) Program, which includes two other housing locations. 

This paper will focus on a CESC program, “The Dwellings” the first tiny home community in the area. The CESC grew out of a church’s effort to provide shelter in the 1980s. By 1991, the Shelter was incorporated and registered as a nonprofit that owned a shelter on West Tennessee Street, where it operated until April of 2015. As the Shelter was unable to provide support services during the day to people experiencing homelessness, a group of community partners, including business owners, social service agencies, and concerned citizens, came together to fill this gap through the Renaissance Community Center (RCC). The RCC provided a place for caseworkers from various agencies to meet with clients in a central location. It was also a place for people to take showers, wash laundry, make phone calls, and use the internet. The Shelter and the RCC worked together to serve the same population and thus shared many clients. Due to a high volume of clients, both locations outgrew their spaces, leading to an agreement to locate their services in a combined building. This combined building, the Kearney Center, opened in April of 2015 [64]. In 2016, the two organizations merged into a single organization, CESC, Inc., the legal entity of the Kearney Center. Since the merger, the CESC has expanded to operate three housing developments: The Kearney Center, Westgate, and The Dwellings. 

These developments aim to address the needs of people at risk of or currently experiencing homelessness. The Kearney Center includes free shelter for the homeless, an onsite clinic, and support services such as General Educational Development (GED) classes, prayer groups, and workforce training. The Kearney Center is considered the “first step in helping stabilize someone who is experiencing homelessness” in the three-rung ladder that CESC operates. 

Westgate is a low-cost housing solution for those either recently experiencing or on the verge of experiencing homelessness. A variety of housing options are offered in order to ease the burden on the individual. This includes a bedroom that is often shared, communal bathrooms, and access to all of the services the Kearney Center provides to clients. Westgate is considered transitional housing and, thus, the second placement step in overcoming homelessness. 

**Figure 1:**
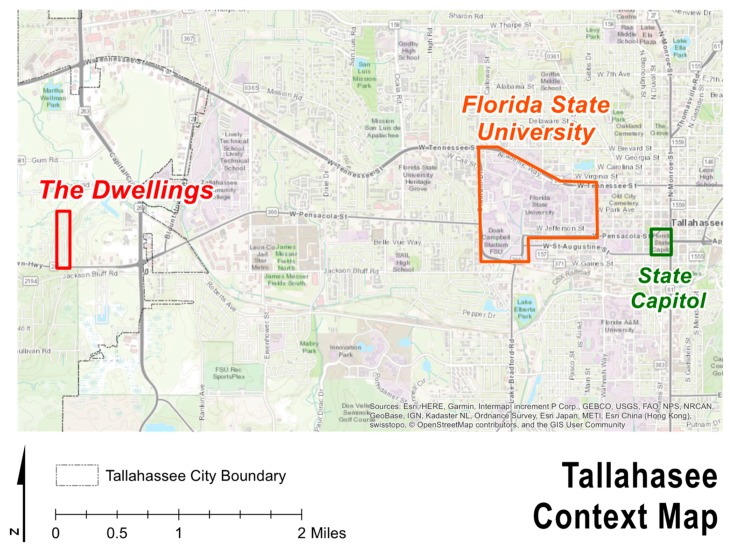
Context Map.

**Figure 2:**
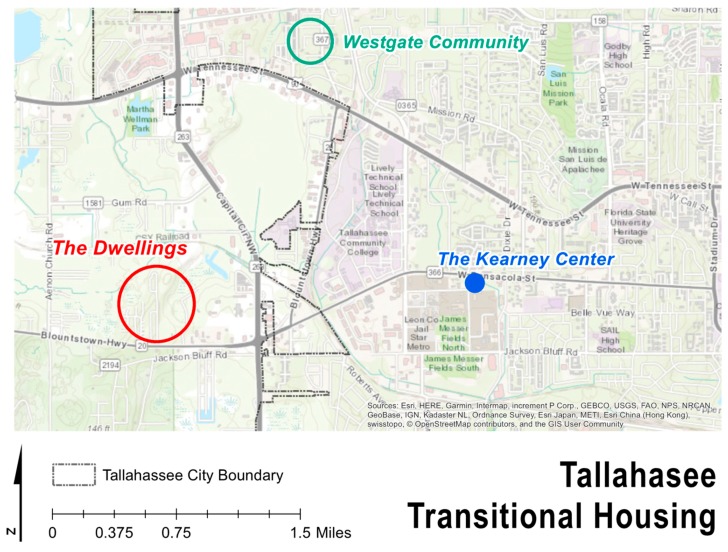
Connecting Everyone to Second Chances (CESC) transitional housing locations.

**Figure 3:**
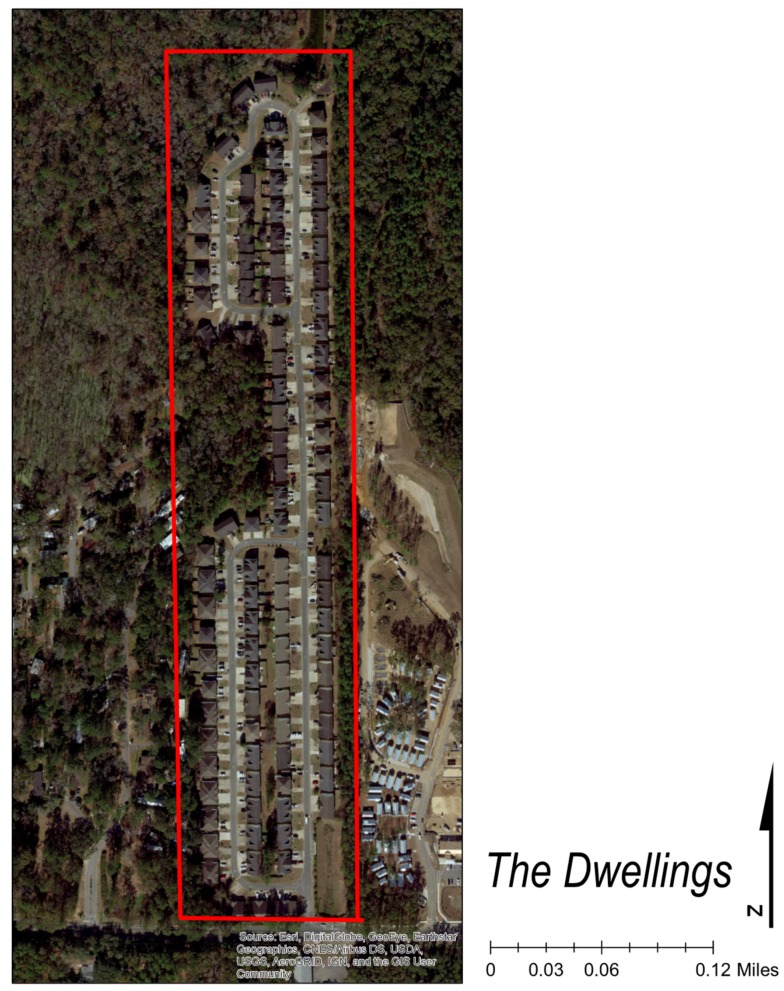
Dwellings Community Plan (Aerial).

The Dwellings is the third and final step in ameliorating homelessness, as well as the first tiny home community in the country dedicated to ending homelessness. The Dwellings opened in December 2017 and offers housing for low- to moderate-income residents in addition to support services [65]. The site is located off Blountstown Highway, on the northwest side of Tallahassee, in Leon County (See Figure 1). Collectively, the physical locations of the CESC’s three housing options are situated in a triangle, providing convenient access to each other. While The Dwellings currently lacks a bus stop near its entrance, there is a circulating shuttle that will take residents between the three sites. Once at the Kearney Center, residents have access to a bus stop and all members are granted a bus pass. There are currently 89 homes in the community with about 10 homes being developed every 45–60 days. By the end of 2019, there is projected to be 130 tiny homes in three floor plans that range from 220 square feet for $600 a month to 410 square feet for $900 a month [66]. At present, the community center and supporting services have recently been completed, and the entire project buildout will include 260 units (See Figure 2 and Figure 3).

## 4. Findings

### 4.1. Process of Participation, Collaboration, and Outreach Efforts

In reviewing The Dwellings’ planning process and outreach efforts, we interviewed CESC staff, residents, and Leon County planners to try to determine the process involved with initiating the development of the tiny home community. Our questions focused on public reaction to the proposal; unexpected barriers to the process; and types of intercommunication between the nonprofits, the County, adjacent neighborhoods, and future residents. We determined through interview and document evidence that there was significant public pushback on the development proposal, making it difficult to obtain a site for the new community, which added an additional hurdle. However, The Dwellings and CESC participation and collaboration into both the planning process and the continued provision of services for clients. Through their extensive outreach and realized success, they managed to mitigate a number of the public’s concerns.

After successfully establishing the Kearney Center and Westgate in Tallahassee, the CESC considered the possibility of establishing a tiny home development to contribute to the founding goals of their organization. They conducted research on the potential for creating a tiny home community for the homeless, and then acquired a property that was being foreclosed just outside the Tallahassee city limits. Yet, once they secured the property and announced their development intentions, there was pushback from surrounding neighborhoods [67]. Nearby residents’ perceptions seemed to be that the new tiny home development would create the same results as the Kearney Center, that is, disrupting traffic, increasing littering, and promoting groups of people gathering on the sides of the roads. A CESC Director further described:

“People were suing [the CESC]. We had so many people in Wolf Creek upset, calling saying like you are going to plummet my property value, I’m never going to be able to sell my house […] we decided ‘I’ll buy it, just what do you want for it and I’ll buy it,’ and we went and bought three houses.”

In order to mitigate these perceptions during the planning and development phases, the CESC participated in outreach strategies that included neighborhood meetings, mass mailings, creating a website, publishing news articles, and speaking with neighbors. A county planner also described the neighborhood resistance to the project:

“That first stage, that was the hardest part because the public was concerned. But once the second part came around, we kept everyone that we emailed at first, all the public, we kept them noticed, so they were on board the second time. They came to the meeting, they had questions, but I think they were more comfortable once they understood what was really being proposed.”

At the same time, county staff described the CESC as very openly communicative and involved throughout their development process. A planner noted:

“They [CESC staff] come in here a lot before they even get started on projects and they’ll talk to us or they have like, they hired a social worker to work with them specifically when they were working with the shelter […] so they’re always trying to be involved with at least the group of folks that they’re working with and I think they’re an advocate for them in a way.”

Over time, concerns were assuaged as the community began to accept the new neighborhood. Standards were developed based on research, experience, and the county’s land development code. While CESC directors have described the entire process as “building this airplane while we fly it”, suggesting both flexibility and spontaneity, many decisions have been made to appease the public. Currently, all of the established tiny homes are occupied and the community center is now built. There is a waitlist to get into The Dwellings, which consists of residents from CESC housing, as well as other applicants. 

### 4.2. Resident Experiences

At the time of our interviews in early 2018, there were over thirty residents living in The Dwellings. Through speaking with staff and residents, we were able to garner an idea of both the community composition and the way in which residents interact with one another. We asked staff questions regarding residents’ backgrounds, how long people generally intended on live in The Dwellings, and community events. In addition to these types of questions, we also asked residents about their experiences so far and how they felt about their neighbors. We found that every resident has a slightly different story on how they ended up in The Dwellings, and subsequently how they feel living there today. The amount of time residents intend on being in the community ranges, though generally some New Urbanist ideas of community-building appear to have been effective in this neighborhood. 

Current residents range broadly in terms of both background and present circumstances. The Dwellings Director explains: “We have residents as young as 9 months old and as old as 80 […] Probably 35–40% have come from either Westgate or the Kearney Center. The rest come from other programs and just the community in general.” As described by The Dwellings staff,

“We do our very best to bring in folks from really struggling to just kind of middle of the road and not so bad. So we have a good mixture, so the community really is synergistic in a sense so that they work with one another, they help one another. If you had 130 people in here that were just completely needy, they couldn’t rely on each other and we couldn’t fulfil all their needs.”

When pushed further on this, The Dwellings Director told us that anecdotal evidence suggests that if you have about 50% of your people that are kind of middle of the road, about 25% anchors, and another 25% that are really struggling, the community seems to work well.

Through speaking about and with residents, this broad range of people became apparent. The Dwellings staff described “A gentleman who’s 71 years of age who lives here, he has a little part-time job driving cars for a rental agency. It’s very fulfilling for him. And in his own words, he’ll probably stay here until he dies.” Alternatively, a middle-aged woman described herself as someone who ended up homeless and found herself staying at the Kearney Center. She waited three months to move into The Dwellings and sees this community as a stopping place on her way back up. As The Dwellings Deputy Director shared, a man in his 40s moved in directly from the Kearney Center and his life crashed and burned in a number of ways:

“Financial, maybe an eviction, I’m not sure all of that, and he found himself homeless at the Kearney Center. Well, he since has found a job and he’s making pretty decent money and he needed to get out of the Kearney Center. He needed a place that was more stable and less, I’m going to use the word chaotic, because if you put 400 people in a building, there’s going to be some chaos. And he has made it here into his own home […] This was like the first step for him in rebuilding his life back to where he needs it to be […] He won’t be here forever. This will be a stopping point for him in his life.”

Regardless of their differences, residents tend to gather in the naturally formed community spaces between the tiny homes. Residents and staff shared stories of community members who get up early in the morning and walk each other to their cars, a couple who carpool to pick up groceries, and those who spend time getting to know their neighbors. 

**Figure 4:**
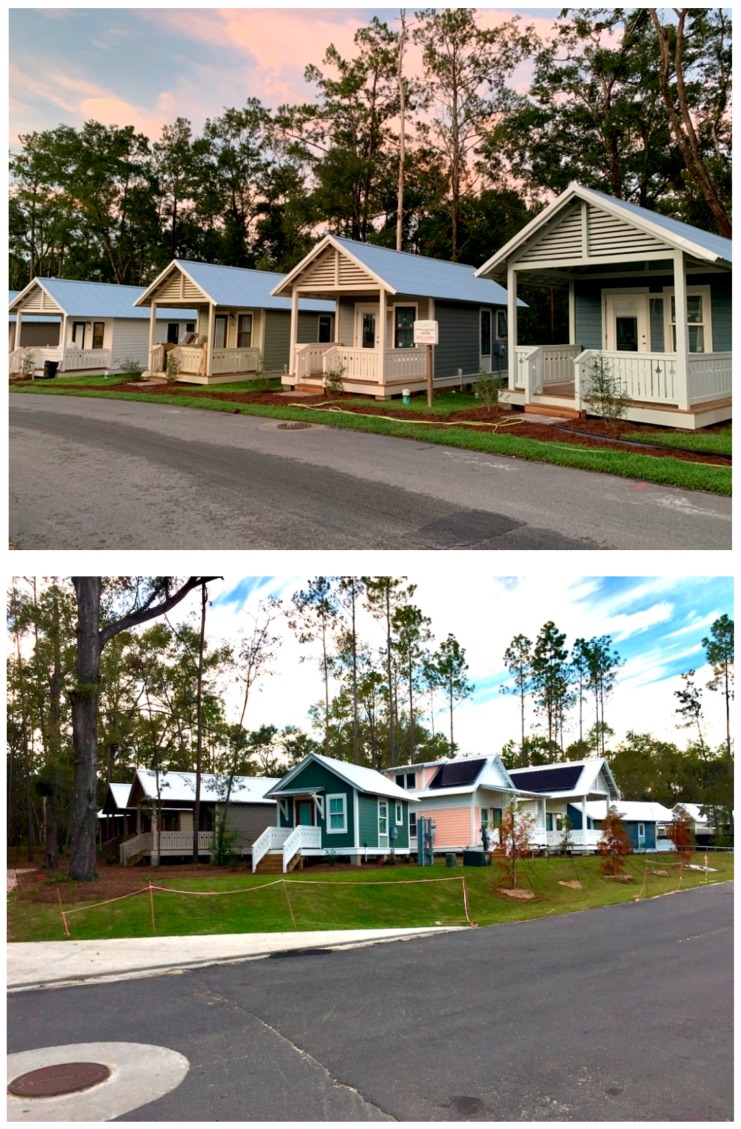
The Dwellings street view.

**Figure 5:**
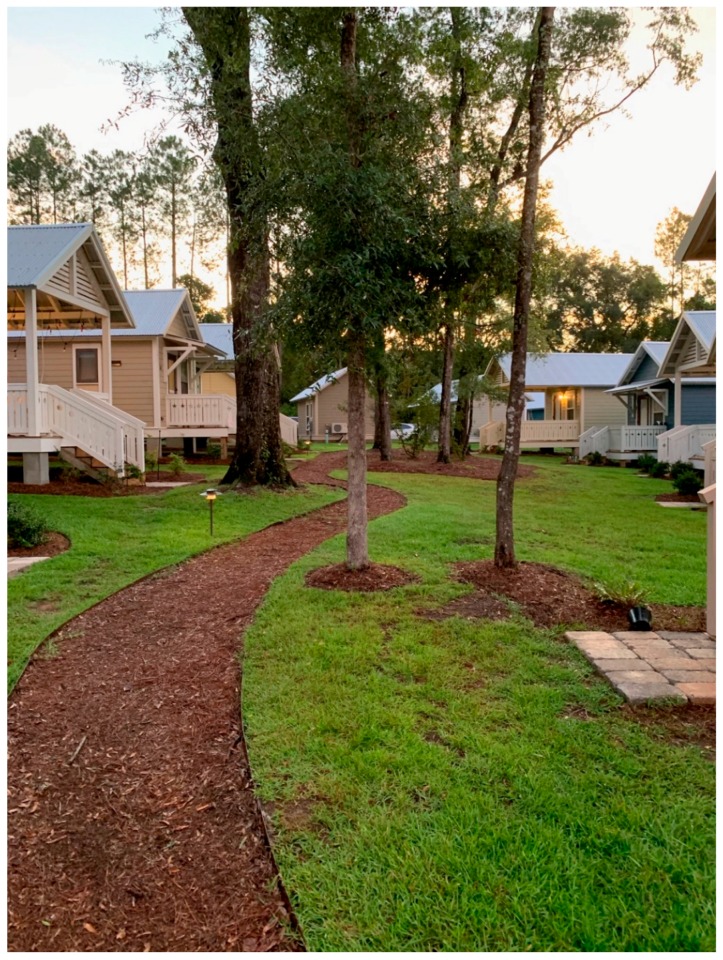
The Dwellings greenway.

**Figure 6:**
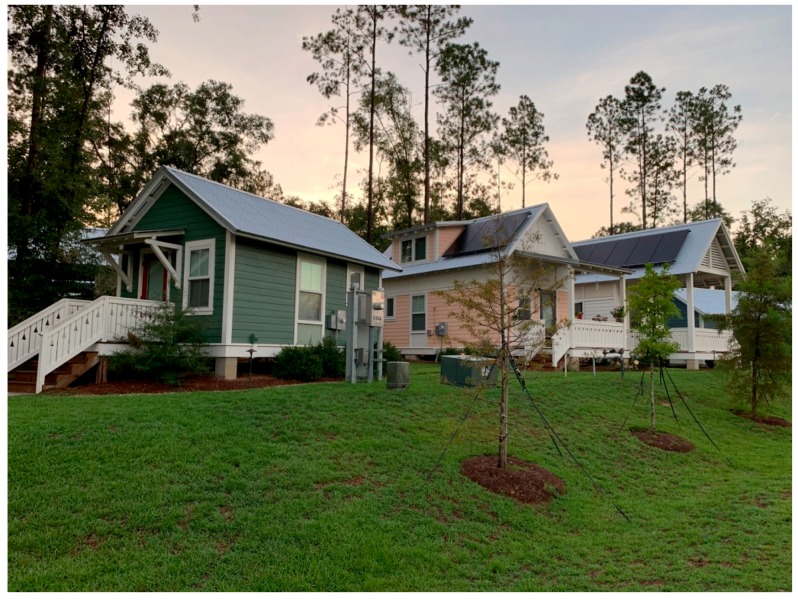

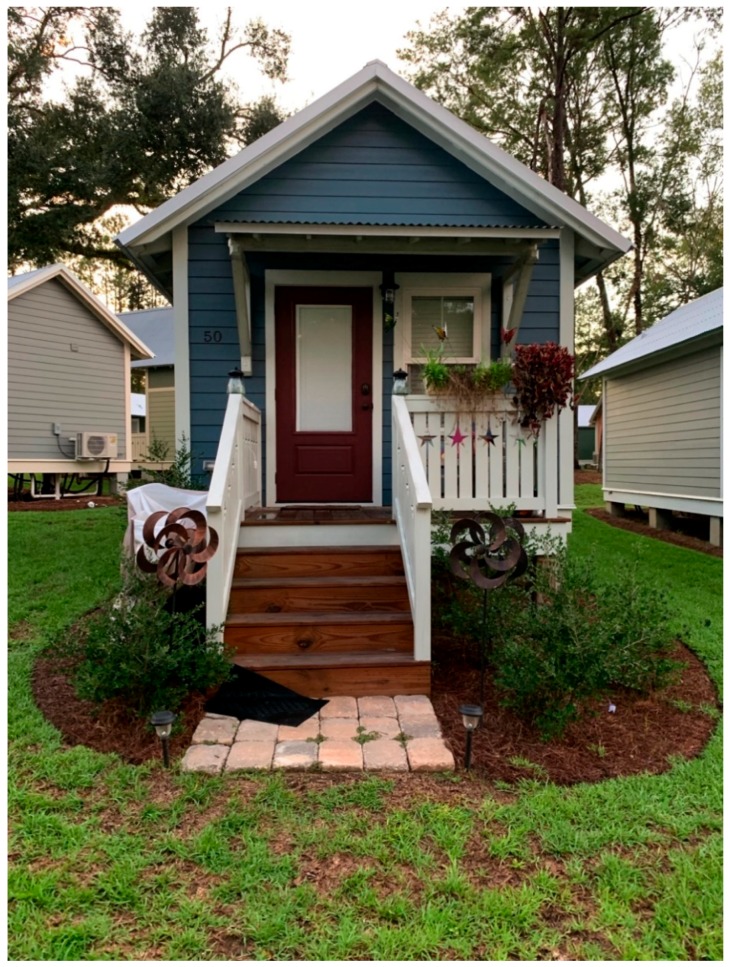
The Dwellings tiny home typologies.

**Figure 7:**
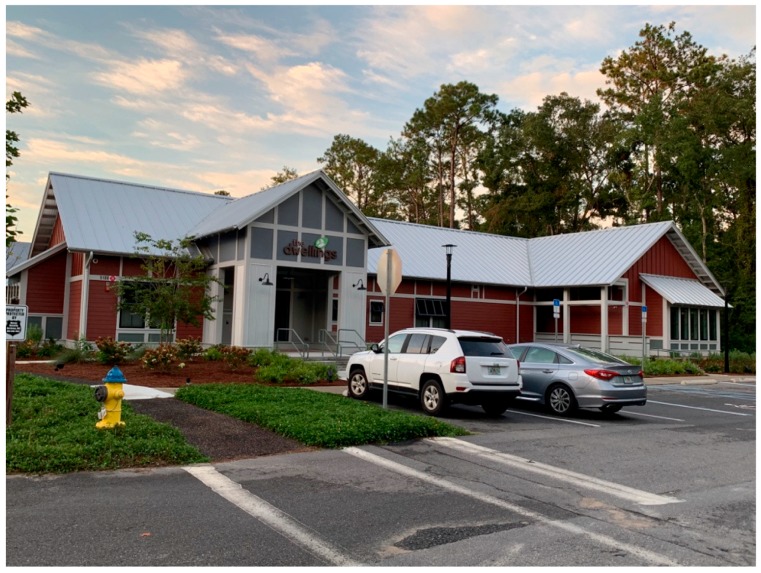

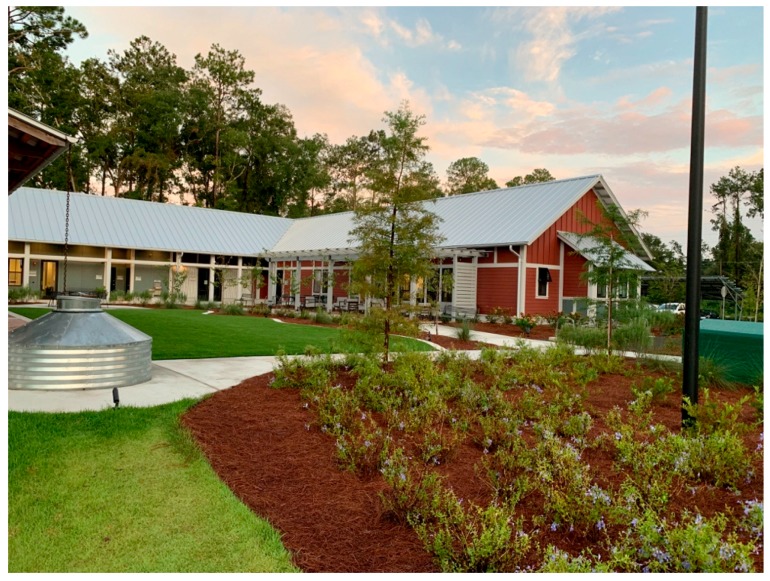
The Dwellings community center.

This seems to align with the aim of The Dwellings to promote the development of social capital and a sense of community. The consultant described the inspiration of The Dwellings’ design as promoting social equity by providing “… a bed to sleep in, a kitchen to cook in, and a public space, in order to have interactions with other people, that became both an internal living space and a front porch.” Each home features a front porch where residents typically gather (See Figure 4, Figure 5, Figure 6 and Figure 7). Thus, The Dwellings internally provides social spaces where residents can gather and get familiar with one another.

However, there are challenges faced by residents, particularly given the isolated location of The Dwellings. A resident expressed concern over the need to walk over half a mile to the nearest bus stop in order to get a ride to work. Similarly, a wheelchair-bound man shared the massive expense of taking a cab ride to the grocery store. While the CESC hopes that a bus stop will eventually be added for the community, this is not guaranteed. The community center provides a small food market and other services. At the time of our interviews, the community center was not yet complete. But even without the community center, residents are planning community events in informal social spaces that create opportunities to interact.

### 4.3. Constraints and Limitations to Implementing The Dwellings

There are constraints and limitations on the implementation of a tiny home community. We asked stakeholders about the challenges faced, especially those which hindered the development, as well as their insights for dealing with these barriers in the future. Funding was a primary issue, as well as NIMBYism (Not in My Backyard-ism), design regulations, equity, and location. 

*Funding.* As noted above, an attribute these communities share is their reliance on non-profit funding. Few of the communities we reviewed were able to rely on more stable sources of income, such as government funding. The Dwellings is entirely supported through a 501(c)(3) corporation, CESC, which was founded through donations and continues to be run on this income source. CESC directors explained how this has provided enough financing to develop the tiny home community:

“[T]he financial resources that it takes are a big stumbling block… [I]n a lot of communities, and actually nationally, they have identified that people who are chronically homeless tend to cost the community a lot more resources… And so they’ve found its cheaper to place somebody in housing, and tiny houses is one of the options that have been tried. And they’ve connected this to HUD funding that helps people stay housed.”

The Executive Director of the CESC reported that The Dwellings is not considered “affordable” housing, and also is unable to receive federal funds from HUD because it is not permanent. In fact, several interviewees shared regret over the need to alter the rental price, and thus the target audience, based on the financial implications of the development as it progressed. The great skill and craft involved in the development of The Dwellings limited the ability to provide lower-cost housing, and this may have restricted the number of people staying at the Kearney Center from becoming residents. The Kearney Center Director lamented “whereas our price isn’t as low as we would ideally want it to be, we do have other low barriers…” These low barriers include extremely limited restrictions in the application process and flexibility of payments. However, several interviewees did express the aim to transition into more affordable units after the first 90 homes are built. This has not yet been finalized and may involve the incorporation of greater density development, such as duplexes, to offset costs. 

### 4.4. NIMBYism

The initial development proposal for The Dwellings resulted in substantial negative community feedback. Unfortunately, homelessness is often perceived as a detractor to neighborhood home values and, thus, the proposal for “tiny homes for the homeless” was contested by adjacent residents. Numerous interviewees suggested barriers in developing tiny homes for the homeless that include financial costs and community concerns. As the Kearney Center Program Director explained, “The biggest barrier is cost and land. Cause you know, you have to go through the City… through a lot of red tape.” NIMBYism manifested through public resistance and protest. The pushback required the CESC to conduct extensive community outreach, fund publications, host meetings, and speak with residents to explain the difference between The Dwellings and a homeless shelter. In the end, residents came around, but this initial hurdle added time and expense to the development process.

### 4.5. Design Regulations

Additional barriers were presented during the physical design of the site. The Dwellings had extensive issues trying to meet code on environmental regulations. As the Dwellings Director explained, there were specific stormwater and drainage requirements that required a holding pond to be built, the drainage on site to be altered, and similar pervious improvements. These kinds of constraints led the CESC to provide more expensive, permeable walkways around the development. The CESC Executive Director added that “the city or the county has expectations that if you call something handicap accessible, it comes with a handicap parking spot, and all kinds of other gradient regulations and everything else gets attached to it.” Essentially, because the site is not a standard suburban development, working around additional requirements that may include parking, stormwater, and permeation was anticipated. This increased time and expenses dedicated to the project.

Although The Dwellings touted an integration of New Urbanism with traditional neighborhood design to appease adjacent communities, based on our interviews, stakeholders acknowledged that there was limited focus on TND elements. While the aforementioned porches may have stemmed from traditional neighborhood design, it is possible that other factors, such as stormwater, outweighed these influences.

### 4.6. Equity

Interviewees also highlighted some of the challenges faced in implementing The Dwellings to provide affordable housing options for those specifically experiencing homelessness. Because rental costs range from $600 to $900 per month (comparable to most housing in Tallahassee), many people facing housing insecurity cannot afford to live here. However, the Kearney Center Program Manager says that “what makes The Dwellings different from any other property in Tallahassee is that it comes along with resources […] the free medical evening clinic, the dentist office, three meals a day, on top of what you have in your household.” This includes 50 partnerships, such as with Tallahassee Community College that allows clients of the Kearney Center to have fees waived, a partnership with StarMetro that provides 30-day bus passes, and a relationship with Bicycle House that will provide members with bikes. The aim of these services is to ensure that residents have affordable housing, employment, healthcare, and a plan moving forward. 

The Dwellings was very deliberate in providing low-barrier housing. Through experiences with clients of the Kearney Center, staff was aware of constant barriers the homeless faced when looking to find housing, as the CESC Executive Director explained:

“Big barriers that we were aware of included the first and last month’s rent, security deposit, and application fee […] Next are eviction histories, credit histories, and criminal histories.”

Many of these things were taken into account when establishing application requirements, and thus many of the people who have these issues will still be able to live in The Dwellings, as described by The Dwellings Housing Manager:

“Sometimes people aren’t gonna pay their fee on time. Again, if you draw a hard line in the sand and say ‘Hey, you’re late or you’re whatever, see ya,’ you’re missing the boat. There’s some benevolence and grace and forgiveness in all of this… If you don’t offer that on the front end than our mission is not complete.”

Yet, the rental process and costs highlight issues of equity in determining who has access to The Dwellings. Residents don’t fill out leases, but rather program applications. In doing this, they aren’t bound to landlord or tenant responsibilities, but are instead held to the standards of the community, which restrict alcoholic consumption and pets, among other things. As the CESC Executive Director explained,

“When they come here, they know full and well that because it’s a program, we have expectation that there’s no prostitution out here, no drug dealing out here, and have we had those things [at the Kearney Center]? Yes. And like do we jump on it immediately? Yes, because we’re protecting the community, we’re protecting you… That’s what we can promise people… our commitment has to be the community and making sure that it’s safe out here.”

This commitment to the community is determined during the program application process and is largely left up to The Dwellings staff to make decisions on who lives in the tiny homes. Before becoming a resident of The Dwellings and entering into the program agreement, one must fill out an application of assessment and have an in-person meeting with the managers of the community to “get an idea of where they are in life and whether or not they would be a good fit for this community,” as described by one of The Dwellings Directors. They use an assessment tool that goes through a number of attributes and evaluates people in regard to their financial, health, and current housing stability. In addition to the assessment tool, decisions are made based on external circumstances. As outlined by staff, “you cannot be a sex offender, you must have an income of some sort, and you must be able to perform activities of daily life.” This process, while open-ended, also serves to support unconscious bias in the selection of residents that “fit” into the ideals of The Dwellings as perceived by staff. If the aim is to provide housing for those experiencing homelessness, a process with more clear guidelines is necessary.

At present, without more affordable options, members of the CESC have had to start evaluating how to deliver more affordable housing options to meet the needs of their intended population. Staff explained this reflective process requires CESC to:

“Begin recognizing where gaps exist, and if we can’t fulfill the needs within those gaps at least being able to speak openly with the community about where those gaps are.”

Given this reflection, the CESC also realizes that The Dwellings can’t serve everyone and, more specifically, they can’t provide housing for some of the clients at the Kearney Center. In addition to this, not everyone would choose to live in a community where residents are required to meet with case managers every week. 

While The Dwellings certainly operates as a low-barrier housing option, there are a number of elements, such as the higher rental cost and selection criteria, that limit its ability to be considered both equitable and a solution to homelessness. As the program continues to develop, there is potential for these issues to be resolved and used as an example of ways to overcome barriers to implementation of future tiny home developments.

### 4.7. Location

Density, mixture of uses, access, and proximity to necessary destinations are highlighted as desired characteristics in traditionally designed urban neighborhoods. Proximity is considered vital because the sustainability of the neighborhood is understood to rely, at least partially, on the residents’ ability to easily access all of the necessary destinations and resources for comfortable living and economic productivity. Unfortunately, in the case of The Dwellings, the discussion on proximity and access come with a significant caveat brought on by the development’s location within the greater Tallahassee area. Considering the location of the development is west of Capital Circle SW, outside the Tallahassee city limits, the development is regionally remote. 

Herein lies one of the primary design issues for The Dwellings, which are not the internal design elements, but rather the larger geographic context. The geographic distance and isolation of The Dwellings is exacerbated by the lack of direct connectivity to Tallahassee-Leon County’s StarMetro bus services. The nearest corridor serviced by StarMetro is east of Capital Circle, and West/Southwest Tallahassee is one of the least-serviced segments of StarMetro’s route network. As The Dwellings Directors confirmed, “The main barrier right now to some of the residents is there is not a StarMetro bus stop right at the front. It’s 0.7 mile away.” Additional barriers exist in terms of residents’ accessibility. To accommodate residents, The Dwellings set out to provide shuttle services for residents. The general consensus among city and county planners interviewed is that it is too soon to pass judgment on the development and whether features such as the location were conclusively detrimental; however, it remains a major point of concern when evaluating the efficacy of the development. 

## 5. Discussion and Conclusions

As tiny home developments are increasing in popularity throughout the United States, communities and organizations are using tiny homes in innovative ways to solve housing insecurity. The Dwellings was utilized as a case study to highlight the inadequacies of offering this affordable housing alternative for those experiencing homelessness. Although intended as a solution to the lack of affordable housing in Tallahassee, The Dwellings exists in a housing market gray area. By definition, it is not considered to be affordable, transitional, or permanent housing. While it was primarily designed to operate for the homeless population that utilizes services at the Kearney Center, the CESC also wanted to create a new urbanist community that could serve as a model program. Through this second goal, costs were higher than anticipated, limiting the level of affordability that could reasonably be achieved. The remaining 130 homes have yet to be designed, which may allow developers some creativity in lowering costs with the aim of serving lower income populations. While this project aimed to provide affordable housing, with costs higher than anticipated, serving those experiencing homelessness has proved to be a more difficult task than intended. Within the broader housing policy arena, this suggests that there are also limitations of local, state, and federal policies that support non-profit affordable housing efforts, which further creates barriers to support low barrier housing.

Despite The Dwellings not currently considered as affordable, the development was very deliberate in providing low-barrier housing. As residents sign a program agreement and have access to a caseworker who helps them develop a plan of success, the likelihood of residents returning to insecure housing seems low. However, The Dwellings is still new, so its efficacy and other outcomes remain to be seen in the long-term. In terms of replication, those involved in The Dwellings development have emphasized the need for flexibility in the design and implementation phases. It may be helpful to hold firm on a price point for units to ensure affordability, or to utilize an “affordable housing” formula so that residents pay 30% of their income based on a sliding scale. This is a measure used by other tiny home and affordable housing initiatives throughout the country. 

Traditional neighborhood design and The Dwellings share many characteristics with regard to density, but it does not appear that New Urbanism was the direct inspiration for density decisions, but rather consequences of the development’s affordability goals. Tiny homes, and more specifically The Dwellings, promote environmental sustainability in more indirect ways with their smaller land needs, reduction of energy use, and design that requires fewer construction materials. High density development occurred through the CESC’s desire to maximize the number of people served. On the other hand, the location of the development hinders mobility. However, city and county planners interviewed acknowledged that it is too soon to draw definitive conclusions about whether the location is detrimental to the project. 

Interviewees were asked about lessons learned and advice for replicating a tiny home development that provides housing for the homeless. An CESC employee stressed the importance of flexibility. A county planner shared that they see tiny homes as a viable option for the homeless if there is sufficient support financially and regulatory compromises at the local level are considered. While Leon County has no tiny home ordinance (they are permitted in single family zoning districts), a planner acknowledged that some municipalities will need to alter their land development codes to permit tiny home communities. Considering the limitations placed on providing the homes at a lower cost, one member of the CESC suggested having a variety of price and size options that align with the range of circumstances experienced by those who are considered homeless or at the risk of becoming homeless. Further, The Dwellings staff shared their ideas around being funded entirely by the government or entirely by a non-profit. The CESC believes that it is essential to have a partnership between the two funding sources to be financially sustainable long-term. Another interviewee suggested the possibility of the project being replicated at the city, local, and state levels. Much else is still being evaluated as the project continues to be built out, but these observations align with our research findings.

Additional research on The Dwellings’ impact on the affordable housing market and homeless issues in Tallahassee is recommended once the development is complete and outcomes are able to be analyzed more thoroughly. Future research may include an examination of the varying backgrounds of residents, the final site and building design, accessibility issues the residents face, as well as the relationship between reconciling issues of housing insecurity and health outcomes for residents. Further, it is possible that, through trial and error, the development may find ways to increase affordability. Should other organizations and cities want to replicate the process, they may face local zoning restrictions and funding problems but can utilize knowledge gained through the CESC’s process of establishing The Dwellings in terms of elements to include in service-based, low-barrier housing communities. 

## Figures and Tables

**Table 1:** Tiny Home Communities Across the United States.

Project	Location	Price/Month	Size	Services	Source of Funding	Target Population	Selection Criteria	Type of Housing
**The Dwellings**	Tallahassee, Florida	$600–$900	220–410 SF	Yes	Non-Profit	On edge or previously Homeless	Application	Varies
**Veterans Community Project**	Kansas City, Nashville, St. Louis	$0	240–360 SF	Yes	Non-Profit	Homeless Veterans	Homeless Veterans	Transitional
**CASS Community Tiny Homes**	Detroit, Michigan	$250–$400	250–400 SF	Yes	Non-Profit	Low-Income	Application	Rental to Homeowner
**Community First! Village**	Austin, Texas	$225–$380	144–200 SF	Yes	Non-Profit	Chronically Homeless Travis County Resident	Application	Permanent Housing
**A Tiny Home for Good**	Syracuse, New York	30% of Income	300 SF	Yes	Non-Profit	Homeless Veterans	Lease Agreement	Permanent Housing
**Infinity Village**	Nashville, Tennessee	$0?	60 SF and 220–400 SF	Yes	Non-Profit	Homeless		Permanent Housing?
**Othello Village**	Seattle, Washington		96 SF and tents		Non-Profit	Households Earning Less than 30% AMI		Transitional Housing
**My Tiny House Project**	Los Angeles, California	Varies	Varies	Yes	Non-Profit	Varies		Transitional Housing
**Second Wind Cottages**	Newfield, New York	Varies	Varies	Yes	Non-Profit	Formerly Homeless Men	Application	Transitional Housing
**The Cottages at Hickory Crossing**	Dallas, Texas	$0? (housing first)	350 SF+	Yes	CDC, non-profits, and gov. (PPP)	Chronically Homeless (who have significant barriers to housing)		Permanent Housing
**Dignity Village**	Portland, Oregon	$20/month	Varies	Yes	City (self- governed)	Homeless Adults (18 y+) with a max capacity of 60 residents	Meeting with Intake Committee: Sweat Equity	Transitional (2 year limit) Housing
**Celebrate Outreach!**	St. Petersburg, Florida	A couple hundred/month	Less than 500 SF	Varies	Non-Profit	Homeless Veterans	Must complete 6 month residential training program	Permanent Housing
